# Food addiction in children: a network analysis of nutritional, metabolic, and sociodemographic factors

**DOI:** 10.1186/s40337-025-01495-5

**Published:** 2025-12-13

**Authors:** Gabriela Carvalho Jurema Santos, André Luiz Góes Pacheco, Tafnes Almeida Oliveira, Isabella Ribeiro Nogueira, Jonathan Manoel Costa, Isabele Goés Nobre, Raquel Canuto, Carol Góis Leandro

**Affiliations:** 1 Federal University of Pernambuco, Academic Center of Vitória, Vitória de Santo Antão, Brazil; 2 Universidade Federal do Rio Grande do Sul (UFRGS), Porto Alegre, Brazil

**Keywords:** Addictive behavior, Food consumption, Children, Complex network, High-order complex network, Nutritional status

## Abstract

**Background:**

Food addiction (FA) is a condition in which ultra-processed foods (UPFs) activate the brain's reward pathways, leading to binge eating, loss of control, and continued consumption despite negative consequences. It can appear early in childhood and is linked to behavioral, sociodemographic, and metabolic factors. This study assessed the contribution of FA, its structure, and connectivity in relation to sociodemographic, nutritional status, and metabolic variables in network analysis.

**Methods:**

A cross-sectional study was conducted with 93 children (7–11 years old) living in Vitória de Santo Antão, Brazil. FA was assessed using the Yale Food Addiction Scale for Children, which was translated and validated for the Brazilian child population. Sociodemographic (age, sex, race, socioeconomic class), anthropometric (body weight, height, waist circumference, BMI, BMI-for-age, body fat percentage, lean mass, and fat mass), and metabolic (blood pressure, total cholesterol, triglycerides, HDL, LDL, and fasting glucose) factors were analyzed. For network analysis, the degree centrality (DC), closeness centrality (CC), betweenness centrality (BC), and eigenvector centrality (EC) were evaluated.

**Results:**

FA exhibited moderate centrality in sociodemographic and metabolic networks, acting as a connector between key variables such as age and socioeconomic class (BC = 0.071–0.500; EC = 0.301–0.500; CC = 0.636–0.667). These metrics indicate that FA, while not dominant, maintains access to influential nodes and participates in relevant information pathways. In contrast, within the anthropometric network, FA showed a peripheral role, with fewer direct links (DC = 0.222–0.285) and limited intermediation (BC = 0.111).

**Conclusion:**

Variation in centrality across domains underscores the selective integration of FA, suggesting that its impact is context dependent.

## Abstract (plain language summary)

Food addiction involves the uncontrolled and impulsive consumption of ultra-processed foods and can occur in childhood. Several factors may be associated with this condition; therefore, this study evaluates the contribution of food addiction within a network analysis framework including sociodemographic, nutritional, and metabolic variables. This study was conducted in Vitória de Santo Antão, Brazil, with children aged 7 to 11 years. Three networks were constructed to analyze the association between food addiction and sociodemographic, anthropometric, and metabolic factors. Food addiction played a moderate role in the sociodemographic and metabolic networks, acting as a link between factors such as age and socioeconomic status. In contrast, in the anthropometric network, it had little influence, appearing more isolated and showing few direct connections with other variables.

## Background

Food addiction (FA) is a behavior related to the excessive and dysregulated consumption of ultra-processed foods (UPF) [1, 2]. This behavior shares characteristics of binge eating and drug addiction, such as loss of control, tolerance, withdrawal, and clinical distress [3]. Worldwide, the frequency of FA in children reaches approximately 15% [4]. During critical periods of development (infancy and adolescence), FA can disrupt the reward system and lead to changes in nutritional status [5, 6].

Changes in nutritional status during childhood among individuals with FA have been associated with an increased risk of developing obesity [7]. Evidence shows that children and adolescents with obesity around the world have a higher frequency of FA (19%) [4]. Thus, the coexistence of obesity and FA can be observed, with obesity representing a well-established risk factor for several metabolic alterations [8]. A longitudinal study with 331 children and adolescents (6–17 years old) demonstrated an association between BMI and an increased risk of unfavorable changes in metabolic variables (total cholesterol, low-density lipoprotein cholesterol, triglycerides, glucose, and systolic blood pressure) [9].

Previous studies have reported that FA appears to be associated with sociodemographic variables, such as age and sex [10, 11]. In a study with Russian adolescents (12–18 years), FA was more prevalent in girls aged 17–18 years [11]. Social determinants also play an important role in the early onset of excessive UPF consumption, especially in low-income regions. In Bangladesh, approximately 83% of adolescents (15.03 ± 0.16) assessed reported consuming at least one UPF the previous day [12]. In northeastern Brazil, approximately 43.70% of children’s (7–10 years) energy intake comes from UPF [13]. These findings show that the prevalence and early onset of FA are influenced not only by physiological factors but also by sociodemographic determinants that influence dietary patterns in childhood and adolescence. This suggests that multiple determinants contribute to both the emergence and consequences of these conditions [14].

Network analysis applies mathematical modeling to capture the complexity of interactions among multiple factors, overcoming the limitations of traditional linear approaches. This methodology enables the identification of central variables, the detection of non-trivial relationships, and the visualization of clustering structures within the studied system. Thus, it constitutes a robust tool for integrating FA with nutritional status, sociodemographic, and metabolic variables, allowing for a more comprehensive understanding of this phenomenon [15].

Using network analysis, we tested the hypothesis that FA is centrally connected to sociodemographic, nutritional status, and metabolic variables. Thus, the main aim of this study was to assess the contribution of FA, its structure, and connectivity to sociodemographic, nutritional status, and metabolic variables in network analysis.

## Methods

### Study and sample description

This is a cross-sectional study, part of the "Growing Healthy" project, conducted in Vitória de Santo Antão, Pernambuco, Brazil, and conducted from March 2022 to July 2023. A city in the northeast, located 46 km from the capital, Recife, with a population of 134,084 inhabitants and a predominantly tropical climate, whose economy is based on vegetables production, and more than 60% of the population is considered brown/black. In addition, it has a low average household income per capita and a low Human Development Index. It has a municipal Human Development Index below the national average (0.640 vs. 0.766 national average), a poverty rate of 49.8%, and a Gini index of 0.42, reflecting social inequality and deprivation in access to education, health, and financial resources [16].

Children and adolescents aged 7–11 years, both sexes, from municipal public elementary schools participated in the study. An a priori power analysis was conducted using the G*Power software (version 3.1.9.7, 2020, Germany) to determine the required sample size. An effect size of 0.6, a statistical power of 0.8, and a significance level of 0.05 were adopted. The test used for the calculation was the independent samples t-test*.* An estimated minimum of 90 participants was required. In addition, for network analysis, a minimum of 10 participants per network node is recommended [17]. Since the largest network in this study included nine variables, at least 90 participants were required.

Schools from different neighborhoods were invited to participate in the study to enable a comprehensive regional mapping. Participation was limited to schools that granted permission, resulting in three schools taking part. The study employed a convenience sampling, with 1,150 participants invited to participate. It included boys and girls aged 7–11 years who were enrolled in the selected schools and had provided informed consent to participate and undergo blood analyses. The exclusion criteria were difficulty answering the questionnaire, inability to perform anthropometric measurements, frequent class absenteeism, participation in quality-of-life and health interventions, and use of medications that interfered with glucose, lipid metabolism, and blood pressure levels. Of the total, only 362 agreed to participate, and only 93 participated in all analyses.

### Food addiction assessment

FA assessment was performed using the Yale Food Addiction Scale for Children (YFAS-C) [18]. This questionnaire has language adapted to the target audience (4–16 years old) and was developed based on seven specific criteria that resemble the symptoms of substance and dependence, as indicated in the Diagnostic and Statistical Manual of Mental Disorders IV (DSM-IV) [19]. The translated and validated version for the Brazilian population was used [20].

The YFAS-C has 25 items divided into a 5-point Likert scale with scores from 0 (never) to 4 (always) (18 items) and dichotomous questions (yes/no) (5 items) that reflect seven symptoms related to addictive eating behavior, distress and clinical impairment: Substance ingested in larger quantities and for longer than intended (items 1, 2, and 3); persistent desire or repeated and unsuccessful attempts to stop (items 4, 17, 18, and 25); too much time/activity to obtain, use, recover (items 5, 6, and 7); important social, occupational, or recreational activities abandoned or reduced (items 8, 9, 10, and 11); continued use despite knowledge of adverse consequences (item 21); tolerance (items 22 and 23); substance taken to relieve withdrawal (items 12, 13 and 14); impairment and distress (items 15 and 16). Items 19, 20 and 24 are not scored for classification purposes. The presence of FA was confirmed when at least 3 symptoms associated with distress and clinical impairment. The YFAS-C was answered individually by the children with the help of the evaluator to avoid embarrassment.

### Assessment of sociodemographic factors

The evaluation of sociodemographic variables was performed using a standardized questionnaire. Age was assessed as a continuous variable (years and months), while sex (girls and boys), race and socioeconomic class were classified as categorical variables. For race, the criteria of the Brazilian Institute of Geography and Statistics (IBGE) were used, which classify individuals by self-observation into white, black, brown, yellow, or indigenous. For socioeconomic criteria, the Brazilian Economic Classification Criteria (ABEP) was used, which estimates the purchasing power of urban people and families and classifies them into A/B1/B2/C1/C2/D/E, with A being the highest level and E the lowest [21].

### Assessment of nutritional status

The nutritional status variables were represented by body weight (kg), height (cm), waist circumference (cm), BMI (kg/m^2^), BMI-for-age (z-score), body fat percentage, lean mass (kg), and fat mass (kg). To assess body weight, a platform scale with a maximum capacity of 150 kg and accuracy of 100 g (Omron, HBF-214, Brazil) was used, and to assess height, a digital ultrasonic stadiometer (Avanutri, AVA-040, Brazil) with a measuring scale with accuracy of 0.1 cm was used. Participants were instructed to perform the procedure standing up, with their backs to the scale, and in an upright position, wearing light clothing. Height was measured between the ground reference plane and the apex, with the individual barefoot. To assess waist circumference, the value measured with an inextensible measuring tape was considered at the midpoint between the anterior superior iliac crest and the last rib, with an accuracy of 0.1 cm. Each anthropometric measurement was performed in triplicate by at least one evaluator. Then, BMI and BMI-for-age were calculated and represented by continuous measurements. To classify nutritional status according to BMI-for-age indicators, age- and sex-specific cutoffs were used, classifying them as eutrophic (z score ≤  + 1) and overweight/obesity (z score ≥  + 1) [22].

To assess body composition (body fat, lean mass, and fat mass), predicted adiposity was estimated using the thickness of the triceps and subscapular skinfolds, using a digital adipometer with 0.1 mm precision (Cescorf®, Porto Alegre, Brazil). Anthropometric measurement techniques followed the recommendations of Lohman et al. (1988), and body fat percentage classification was in accordance with the criteria of Slaughter et al., (1988) and Lohman and Going, (2006).

### Assessment of and metabolic variables

Systolic blood pressure (SBP) and diastolic blood pressure (DBP) were measured by the auscultatory method, using a pediatric stethoscope and aneroid sphygmomanometer (Premium®, Medical Instruments, China), with a measurement range between 0 and 300 mmHg, previously calibrated, selecting the cuff of the appropriate size for the child's arm. All blood pressure measurements were performed and classified following the recommendations adopted by the Brazilian Guidelines for Arterial Hypertension (BARROSO et al. 2021).

To assess the biochemical profile, blood was collected between 7 and 9 a.m., after a 10- to 12-h fast. The lipid profile was measured using total cholesterol (TC), triglycerides (TG), high-density lipoprotein (HDL), low-density lipoprotein (LDL), and fasting glucose. The analysis was performed using the Cholestech LDX device (Hayward, CA, United States) (FRIEDEWALD; LEVY; FREDRICKSON, 1972).

### Mathematical modeling

**Fig. 1:**
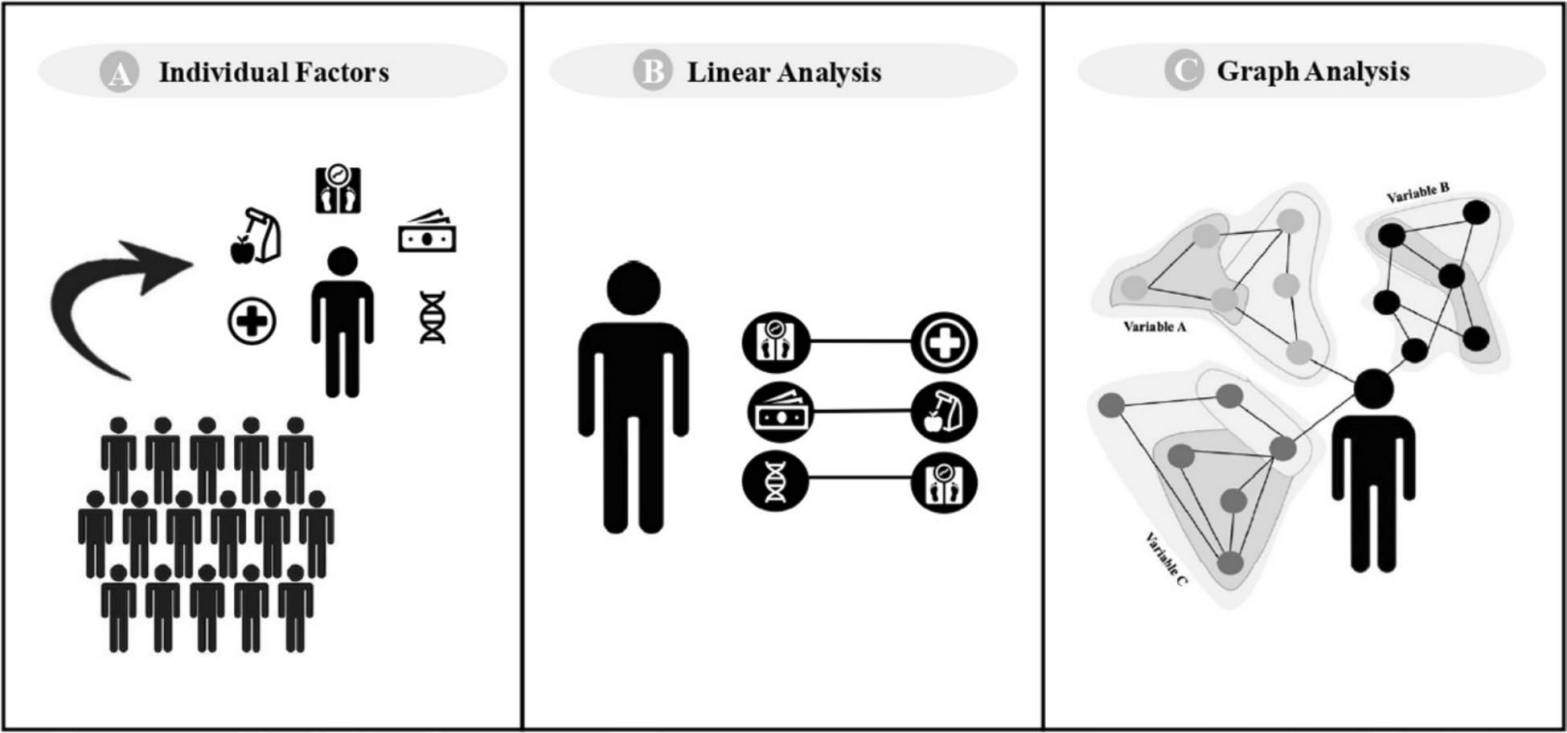
Individual factors and analytical approaches: linear models versus complex networks. **A** Traditional studies often focus on individual responses and their associated phenomena within the population. The characterization of an individual, however, involves multiple dimensions, including physiological, social, and behavioral factors, forming a complex system. **B** Linear analyses, such as correlations and linear regressions, focus on the isolated effects of variables on individuals, failing to assess the complex and interconnected nature of health phenomena. **C** Graph analysis allows for the integration of multiple variables, revealing patterns of interaction that go beyond isolated associations. This approach provides a more comprehensive understanding of the complexity of individuals and how these interrelationships influence health outcomes

Data was entered in Epidata program® (version 3.1, 2011, Denmark). The network analysis was conducted to assess the centrality of FA and its connections with sociodemographic, nutritional status, and metabolic variables on the in a Python library® (version 3.13, 2024, United States of America). To this end, this study employed an approach based on graph theory (Fig. 1). In this context, a graph (G) is a mathematical structure used to represent relationships, consisting of vertices (or nodes, i.e., variables) and edges (i.e., connections or relationships between variables). A graph is defined as G = (V, E), where V denotes the set of nodes (or vertices) and E ⊆ V × V represents the set of edges, that is, the pairwise relationships between nodes [23].

However, graphs can capture only dyadic interactions, limiting their ability to represent multidimensional group relationships. To overcome this limitation, we adopt a hypergraph structure that allows simultaneous representation of connections among two or more elements. This enables the modeling of multidimensional or multivariate interactions. A hypergraph can be defined as H = (V,E), where V = {v1, v2, …, vn} is the set of nodes and E = {e1, e2, …, em} is the set of hyperedges (a connection that simultaneously joins two or more vertices), with each hyperedge e_m_ ⊆ V connecting an arbitrary number of nodes simultaneously [23, 24]. This generalization allows hypergraphs to capture higher-order and polyadic interactions, involving three or more elements simultaneously, that cannot be represented in simple graphs.

In this study, the correlation matrices were constructed and subsequently used to generate hypergraphs (implemented in Python with NetworkX and HyperNetX) for each group of interest: FA by demographic profile (threshold 0.2), FA by nutritional status (threshold 0.2), and FA by metabolic profile (threshold 0.3). The compositions of the resulting hyperedges were then analyzed, and four centrality measures, such as, the degree (DC), the closeness (CC), the betweenness (BC), and the eigenvector centrality (EC) were computed for each vertex [23].

BC identifies how often a variable appears on the shortest paths between two other variables, acting as a bridge for information flow across different parts of the network. DC measures the number of direct connections that a variable has with others, reflecting its connectivity and potential influence within the network. EC assesses the importance of a variable based on the influence of the variables to which it is connected, thus considering the quality of its connections. Finally, CC evaluates the average distance of a variable to all other variables, indicating its ability to quickly interact with other parts of the network. Centrality values ≤ 0.3 were considered weak, between 0.4 and 0.6 were considered moderate, while values ≥ 0.7 were considered strong. From the centrality analysis, it is possible to identify whether a variable plays a central role (high connectivity and crucial influence within the network) or a peripheral role (less influence but able to connect different parts of the network) [25].

This procedure enables a detailed evaluation of the role played by individual variables within the multidimensional network and, by analyzing hyperedge compositions, reveals the collective influence of multiple variables acting together. This approach has been used in previous studies, particularly in contexts dealing with complex psychiatric phenomena [26, 27]. Accordingly, the numerical analysis of hypergraph centralities and their contextual interpretations provides novel insights into group-level behaviors of interacting variables that may underlie the emergence of FA [28].

Two primary dimensions were evaluated: individual node centrality, which assesses the connections between pairs of variables, analyzing the position and influence of each variable individually within the network, and the structure of hyperedges connecting multiple variables, which allows for observing interactions among multiple elements simultaneously, evaluating the collective structure and interaction patterns within groups through hyperedges that connect more than two variables at the same time [29].

The associations between variables were calculated using a mixed-type correlation matrix that automatically adapts to the variable types. Specifically, Pearson’s correlation coefficient (r) was applied for continuous variables, Cramér’s V for categorical variables, and eta-squared (η^2^) from one-way ANOVA for continuous-categorical variables. Before constructing the network and hypergraph, we examined redundancy using pairwise correlations in a mixed-type matrix to control for multicollinearity. In cases where |ρ|≥ 0.90, the pair of variables was removed before network and hypergraph analysis.

To evaluate the stability of the hypergraph centralities**,** a nonparametric bootstrap analysis was performed. A total of 500 bootstrap replicates were generated, in which the rows and columns of the association matrix were randomly resampled with replacement. For each replicate, the hypergraph was reconstructed, and the four node centrality measures were recomputed. Stability was quantified using the Spearman correlation between the original and bootstrapped centrality values, which served as the stability index.

### Statistical analysis

Statistical analyses were performed STATA software® (version 16, 2019, United States of America). Categorical data were presented as absolute number (N) and relative frequency (%), and continuous variables were expressed as mean and standard deviation, or median and minimum and maximum values, depending on the data’s normality. A 95% confidence interval (95% CI) was adopted. Data normality was tested using the Kolmogorov–Smirnov test. Chi-square, student t-test, and Mann–Whitney tests were used to assess differences between groups. To assess the internal consistency of the YFAS-C, Cronbach’s alpha was calculated.

## Results

The description of the socioeconomic variables, frequency of FA, anthropometry, and metabolic factors are described in Table 1. Of the included participants, 54.9% (n = 51) were male, with a mean age of 9.13 ± 1.0 years old. This was the only variable showing a difference between genders, with girls having a higher mean age. Regarding FA, a frequency of 25.8% (n = 24) was observed, with no difference between genders. As for the other variables, no differences were observed between genders. The YFAS-C showed a Cronbach’s alpha coefficient (α) of 0.78, indicating good reliability of the instrument.

**Table 1:** Description of frequency of food addiction, sociodemographic, anthropometric and metabolic variables of children and adolescents (n = 93)

	Girls (n = 42)	Boys (n = 51)	95% CI	*P* value
N (%)				
Food addiction, n (%)			0.17–0.35	0.176
Yes	8 (19.0)	16 (31.4)		
No	34 (81.0)	35 (68.6)		
Race, n (%)			2.21–2.56	0.716
White	8 (19.0)	11 (21.6)		
Black	11 (26.2)	10 (19.6)		
Brown	23 (54.8)	30 (58.9)		
Socioeconomic class, n (%)			1.41–1.62	0.125
B/C	24 (57.1)	21 (41.2)		
D/E	18 (42.9)	30 (58.8)		
Nutritional status, n (%)			1.28–1.48	0.071
Eutrophic	22 (52.4)	36 (70.7)		
Overweight/obesity	20 (47.5)	15 (29.4)		
Mean (SD) or median (min.–max)				
Age	9.1 ± 0.9	9.0 ± 1.1	8.9–9.3	0.024
Weight (kg)	32.4 (20.1–76.6)	30.5 (18.2–65.8)	32.3–36.9	0.394
Height (cm)	135.9 ± 9.3	136.7 ± 9.6	134.3–138.2	0.521
Waist circumference (cm)	63.0 (52.0–98.0)	61.0 (47.0–93.0)	62.2–66.7	0.146
BMI (kg/m^2^)	17.3 (13.5–32.8)	16.8 (11.5–29.0)	17.1–19.1	0.247
BMI-for-age (z-score)	1.0 ± 1.6	0.3 ± 1.3	0.3–0.9	0.127
Body fat (%)	19.1 (10.1–43.9)	20.9 (9.1–42.8)	20.4–24.2	0.924
Lean mass (kg)	26.9 ± 5.5	25.5 ± 5.1	25.1–27.3	0.996
Fat mass (kg)	6.0 (0.0–33.6)	6.2 (0.0–28.2)	7.0–9.8	0.932
Total cholesterol (mg/dL)	142.5 ± 26.7	140.8 ± 27.9	136.1–147.3	0.921
HDL-cholesterol (mg/dL)	42.2 ± 14.0	37.6 ± 12.2	36.9–42.4	0.659
LDL-cholesterol (mg/dL)	85.0 (46.0–307.0)	81.5 (37.0–141.0)	81.4–85.3	0.823
Triglycerides (mg/dL)	70 (45.0–192.0)	70 (45.0–178.0)	70.8–84.9	0.639
Fasting blood glucose (mg/dL)	89.2 ± 7.2	83.6 ± 6.2	84.6–87.6	0.934
Systolic blood pressure (percentile)	50 (8.2–121.2)	49.8 (11.6–70.7)	47.8–53.2	0.452
Diastolic blood pressure (percentile)	97.1 (63.0–140.0)	98.6 (72.0–146.6)	98.1–105.1	0.714

BMI, body mass index; HDL, high density lipoprotein; LDL, low density lipoprotein; SD, standard deviation. Differences assessed using the χ^2^ test for categorical variables, Student’s t-test for means, and Mann–Whitney test for medians

The stability of the hypergraph centrality measures was quantified using the Spearman’s correlation between the original values and those obtained from the bootstrap replicates. The results indicated a mean stability (rₛ) of 0.25 ± 0.28, with a median of 0.27 and a range from − 0.63 to 1.00 (n = 998 replicates), suggesting low-to-moderate stability. When analyzed individually, the four centrality measures showed median correlations close to 0.5, indicating that the node-ranking structure remained largely preserved across resamples, reflecting moderate centrality stability and consistency with previous empirical findings in mixed-type hypergraphs with small sample sizes (Table 2). A sensitivity analysis employing mean imputation demonstrated that the hypergraph centrality measures remained stable, with negligible changes observed (Δ < 0.01). Missing data were addressed prior to hypergraph construction. A complete case analysis was conducted for continuous variables. For binary variables, missing values were replaced with zero when this indicated the absence of the condition.

**Table 2:** Stability of the hypergraph centrality measures quantified using Spearman correlation coefficients

Centrality	Mean rₛ	SD	Median	P25	P75	Min	Max	n	Interpretation
Degree	0.42	0.41	0.50	0.19	0.74	– 0.94	1.00	500	Moderate stability
Betweenness	0.37	0.49	0.49	0.03	0.73	– 0.94	1.00	500	Low–moderate stability
Closeness	0.44	0.38	0.51	0.22	0.73	– 0.94	1.00	500	Moderate stability
Eigenvector	0.44	0.39	0.49	0.23	0.77	– 0.89	1.00	500	Moderate stability

Mean r_s,_ average of Spearman's correlation coefficient; SD, standard deviation; P25, 25th percentile; P75, 75th percentile; Min, minimum; Max, Maximum, n, number of observations

### Analysis of centrality metrics

**Fig. 2:**
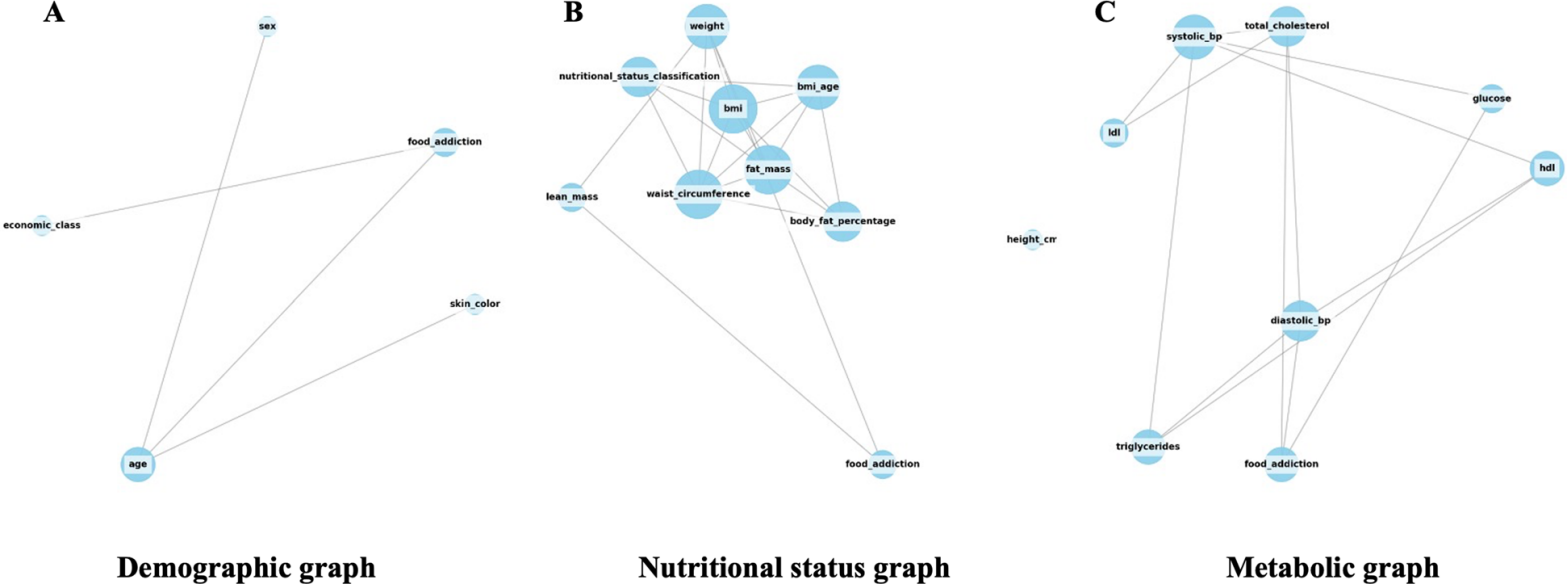
Graph networks representing food addiction in relation to sociodemographic variables (**A**), nutritional status (**B**), and metabolic status (**C**). A threshold of 0.5 was applied to visualize the connections. *BMI, body mass index; LDL, low density lipoprotein; HDL, high density lipoprotein*

The graphs representing the networks among FA, sociodemographic variables, and nutritional and metabolic status are shown in Fig. 2. A first network was constructed connecting FA with sociodemographic variables (sex, race, and socioeconomic class) (Table 3). In this structure, age emerged as the most central node, with high intermediary potential (BC = 0.833), strong direct connectivity (DC = 0.750), high-quality links (EC = 0.653), and network accessibility (CC = 0.800). Race and socioeconomic class were associated with lower levels of connectivity and influence. FA, although not dominant, demonstrated a moderate role as a connector (BC = 0.500), with intermediate influence (EC = 0.500) and access to other variables (CC = 0.667).

**Table 3:** Analysis of centrality metrics

	Betweenness centrality	Degree centrality	Eigenvector centrality	Closeness centrality
Food addiction and sociodemographic				
Age	0.833	0.750	0.653	0.800
Race	0.000	0.250	0.353	0.500
Socioeconomic class	0.000	0.250	0.270	0.444
Food addiction	0.500	0.500	0.500	0.666
Food addiction and nutritional status				
Weight	0.333	0.555	0.280	0.646
Height	0.000	0.000	0.000	-
Waist circumference	0.090	0.666	0.428	0.711
BMI	0.090	0.666	0.428	0.711
BMI-for-age	0.006	0.555	0.382	0.547
Body fat	0.000	0.444	0.328	0.507
Lean mass	0.000	0.222	0.068	0.418
Fat mass	0.090	0.666	0.428	0.711
Food addiction	0.000	0.222	0.068	0.418
Food addiction and metabolic				
Total cholesterol	0.134	0.571	0.410	0.700
HDL-cholesterol	0.015	0.428	0.353	0.636
LDL-cholesterol	0.000	0.285	0.251	0.583
Triglycerides	0.015	0.428	0.353	0.636
Fasting blood glucose	0.023	0.285	0.219	0.583
Systolic blood pressure	0.309	0.714	0.459	0.777
Diastolic blood pressure	0.142	0.571	0.410	0.700
Food addiction	0.071	0.428	0.300	0.636

BMI, body mass index; HDL, high density lipoprotein; LDL, low density lipoprotein

In the second network, which included FA and nutritional status variables (weight, height, waist circumference, BMI, BMI-for-age, body fat, lean mass, and fat mass) (Table 3), body weight stood out as a relevant connector (BC = 0.333), with several direct connections (DC = 0.556) and moderate accessibility (CC = 0.646). Waist circumference, BMI, and fat mass showed limited intermediary roles but high connectivity (DC = 0.667), structural influence (EC = 0.428), and network proximity (CC = 0.711). In contrast, height, BMI-for-age, lean mass, and FA had low centrality across all metrics, indicating a peripheral position. Thus, FA plays a secondary role in this network, with weak integration into anthropometric relationships.

Finally, a third network linked FA to metabolic variables (total, HDL, LDL cholesterol, triglycerides, fasting glucose, and systolic and diastolic blood pressure) (Table 3). SBP was the most central node (BC = 0.309; DC = 0.714; EC = 0.459; CC = 0.778), followed by DBP and total cholesterol, which showed moderate relevance (BC ≤ 0.143; DC = 0.571; EC ≤ 0.410; CC = 0.700). FA, along with HDL and triglycerides, exhibited peripheral roles (BC ≤ 0.071), though with moderate structural influence (DC = 0.428; EC ≤ 0.353) and high communication potential (CC = 0.636). These values suggest that FA, although not structurally central in the metabolic network, remains integrated and able to interact with key variables.

### Multidimensional hypergraph analysis

The node centrality assessment explores how each variable individually contributes to the connectivity and influence within the network. FA did not occupy a central position in the sociodemographic or metabolic networks, with low betweenness centrality (BC ≤ 0.071) and moderate direct connectivity (DC = 0.500), indicating a peripheral yet integrated variable. Despite not being a structuring node, FA exhibited a moderate capacity to influence other variables (EC = 0.500) and maintain network accessibility (CC = 0.667), particularly through its links with age and socioeconomic class (Table 4).

**Table 4:** Multidimensional hypergraph analysis

	Betweenness centrality	Degree centrality	Eigenvector centrality	Closeness centrality
Food addiction and sociodemographic				
Age	0.166	1.000	0.510	1.000
Race	0.000	0.750	0.439	0.800
Socioeconomic class	0.166	1.000	0.510	1.000
Sex	0.000	0.750	0.439	0.800
Food addiction	0.000	0.500	0.306	0.666
Food addiction and nutritional status				
Weight	0.022	1.000	0.335	1.000
Height	0.000	0.556	0.201	0.692
Waist circumference	0.022	1.000	0.335	1.000
BMI	0.000	0.888	0.314	0.900
BMI-for-age	0.000	0.888	0.314	0.900
Body fat	0.000	0.888	0.314	0.900
Lean mass	0.022	1.000	0.335	1.000
Fat mass	0.022	1.000	0.335	1.000
Food addiction	0.022	1.000	0.335	1.000
Food addiction and metabolic				
Total cholesterol	0.095	0.714	0.408	0.777
HDL-cholesterol	0.079	0.571	0.325	0.700
LDL-cholesterol	0.047	0.571	0.331	0.700
Triglycerides	0.055	0.571	0.327	0.700
Fasting blood glucose	0.055	0.571	0.327	0.700
Systolic blood pressure	0.079	0.714	0.408	0.777
Diastolic blood pressure	0.055	0.571	0.327	0.700
Food addiction	0.055	0.571	0.327	0.700

BMI, body mass index; HDL, high density lipoprotein; LDL, low density lipoprotein

In the nutritional status network, FA showed lower centrality (DC = 0.222–0.285), contradicting earlier suggestions of centrality. Although its closeness centrality was relatively high (CC ≥ 0.666), its betweenness and eigenvector values remained low (BC ≤ 0.111; EC ≤ 0.335), reinforcing its peripheral role in relation to anthropometric variables (Table 4).

## Discussion

The present study aimed to evaluate the relative importance of FA, its structure, and connectivity in relation to sociodemographic variables, nutritional status and metabolic variables within then network. The network structure enable the identification of connection patterns among variables and the role of FA within the complex interplay of risk factors and systemic influences. Our hypothesis was that FA is centrally connected to sociodemographic, nutritional status and metabolic variables in the network analysis. However, FA played a peripheral role in networks with sociodemographic, nutritional status, and metabolic variables.

As expected, sociodemographic, nutritional status, and metabolic variables were strongly interconnected in the network. This confirms that network analysis, based on graph theory, can be a reproducible approach in clinical studies, allowing the modeling and understanding of complex interactions between variables and health outcomes [30].

Contrary to our hypothesis, FA played a peripheral role in the sociodemographic network, although it is related to age and socioeconomic status. In this context, it demonstrated a moderate role in connecting variables (BC), with moderate influence (EC) and good accessibility (CC), indicating that it can affect and interact with other variables efficiently. In a sample of 6,587 Australian adolescents (aged 10 to 14), when assessed linearly, gender was associated with FA, with females being a strong predictor [31]. In Iranian children, FA was also associated with gender, but in this case with males. An association with age over 8 years was also observed. No significant associations were found between FA and socioeconomic status or parental education [32]. In a study of Brazilian public school children aged 7 to 12, FA was not associated with age [33].

Although these studies highlight important associations between FA and sociodemographic variables across different contexts, they rely on traditional analytical approaches that fail to capture integrated connections. Given that the onset of FA is associated with the consumption of UPFs, it is important to consider the influence of social context [34]. Areas characterized as food deserts or food swamps, typically located in low-income communities, tend to offer a predominance of UPFs options [35]. This disproportionate availability increases exposure to such products, exacerbating the risk in socially vulnerable populations [35].

Given that FA is influenced by multiple domains, the present study reinforces the value of network analysis for understanding the complex interactions among these factors. Unlike previous approaches, this method offers greater sensitivity by integrating variables from diverse domains, such as sociodemographic and psychobiological, thereby contributing to a more comprehensive understanding of the individual as a complex system [36].

In the network analysis of nutritional status variables, FA occupied a peripheral position, as it presented low connectivity (BC), few connections (DC), limited influence (EC), but demonstrated a high capacity for interaction with other variables (CC). A previous study found that FA was not associated with anthropometric measures, including weight, height, BMI-for-age, or height-for-age in Brazilian children aged 9 to 11 [37]. However, other studies have demonstrated positive associations between FA and indicators of nutritional status [7, 32]. Naghashpour et al. [32] observed, in a cross-sectional study of 222 children aged 7 to 13 years in Ahvaz, Iran, that FA was correlated with BMI and BMI z-scores in girls. In boys, associations were not observed [32]. In another study involving Chinese children aged 8–17 years, FA was positively correlated with BMI z-scores and waist-to-height ratio, which was used to assess weight status [7].

Furthermore, FA has a positive and moderate effect on obesity and significantly increases the risk of obesity in children [38, 39]. However, our results demonstrated weak connection with nutritional status variables. Therefore, FA may not manifest directly through common body indicators, highlighting the need for psychological and behavioral assessment tools, in addition to anthropometric data, for its diagnosis.

In the network of metabolic variables, FA is not at the center of metabolic regulation, but it is part of the network (DC = 0.428; EC ≤ 0.353) and has the potential to affect or be affected by other metabolic conditions (CC = 0.636). In Brazilian children aged 7 to 16 years with a double burden of malnutrition, FA symptoms were associated with higher body fat and increased levels of insulin and leptin [5]. On the other hand, in the same population (Northeast Brazil), FA in children aged 7 to 12 years was not associated with total cholesterol, HDL, LDL, triglycerides, fasting glucose, insulin, or HOMA-IR [33]. However, little is discussed about the repercussions of FA and metabolic outcomes in childhood; this is the first study to report a network connection between FA and metabolic markers in childhood. Furthermore, the peripheral connection between FA and metabolic variables may be an outcome of the relationship with obesity [40].

This study has some limitations. First, it used convenience sampling and did not assess the family context. Nevertheless, the network analysis methodology is adaptable to different clinical settings, enhancing the reproducibility of results and supporting the development of predictive models, while remaining sensitive to confounding variables. Moreover, it acknowledges that, beyond physiological factors, social determinants of health should also be targeted in interventions to promote and protect health. Second, FA was assessed using a self-report scale. However, this remains the only available method for evaluating this outcome, highlighting the need for the development of more sensitive tools in the future. Third, this is a cross-sectional study, which limits the ability to draw causal inferences. Fourth, the sample size of 93 participants, although within recommended guidelines, may lead to underestimation of network connectivity or overestimation of network fragmentation, limiting the ability to draw generalizable conclusions about the network’s structure or dynamics. Fifth, using a child population may introduce self-report bias and fail to control for cognitive maturity. However, the YFAS-C was specifically designed to be completed by children themselves, using simplified, age-appropriate language.

This study contributes to FA research by offering directions for future investigations into its causal pathways and associations with environmental factors, including social, anthropometric, and metabolic aspects. This work advances the field by providing a more integrated and nuanced understanding of the multiple domains involved in FA. Unlike traditional linear methods that examine variables in isolation, network analysis reveals how they interact within a broader system, identifying central elements and interconnectivity patterns that might otherwise go unnoticed. Network analysis is a tool that allows the identification and interconnection of variables within a system, providing guidance for the planning of potential interventions. In the context of the present study, the links between FA and variables such as age and socioeconomic status highlight the importance of schools and the family environment as central elements in health promotion. These findings also enable identification of key points in the community environment that could be targeted for strategic change. This approach supports the development of more targeted interventions focused on key factors linked to FA and enables the identification of distinct risk profiles based on these patterns.

## Conclusion

In conclusion, although FA does not constitute a central variable, it showed a moderate capacity to influence other variables, as well as high accessibility, particularly about factors such as age and socioeconomic status. This finding underscores the potential role of environmental and contextual factors in the development of FA during childhood. Furthermore, its weak association with common indicators of nutritional status, such as BMI, suggests that FA may not be directly reflected in traditional anthropometric measures.

This study underscores the importance of considering FA within a broader biopsychosocial framework when evaluating children’s metabolic and nutritional health. Clinically, it highlights the need for comprehensive assessments that go beyond traditional measures like BMI to include sociodemographic, behavioral, and psychological factors, showing that FA should not be overlooked even in the absence of clear anthropometric or metabolic changes. For public policy, the findings underscore the importance of primary prevention strategies that address socioeconomic factors, particularly during childhood, and consider the environments in which children are embedded, such as schools, families, and communities.

## Data Availability

The datasets used and/or analysed during the current study are available from the corresponding author on reasonable request.

## Abbreviations

FA: Food addiction
UPF: Ultra-processed foods
YFAS-C: Yale Food Addiction Scale for Children
IBGE: Brazilian Institute of Geography and Statistics
DSM-IV: Diagnostic and Statistical Manual of Mental Disorders IV
ABEP: Brazilian Economic Classification Criteria
TC: Cholesterol
TG: Triglycerides
HDL: High-density lipoprotein
LDL: Low-density lipoprotein
SBP: Systolic blood pressure
DBP: Diastolic blood pressure
BC: Betweenness centrality
DC: Degree centrality
EC: Eigenvector centrality
CC: Closeness centrality
CI: Confidence interval

## References

[1] Jurema Santos GC, de Sousa Fernans MS, Carniel PG, da Silva Garcêz A, Góis Leandro C, Canuto R. Dietary intake in children and adolescents with food addiction: a systematic review. Addict Behav Rep. 2024;19:100531. 38322322 10.1016/j.abrep.2024.100531 PMC10844934

[2] Gearhardt AN, Schulte EM. Is food addictive? A review of the science. Annu Rev Nutr. 2021. 10.1146/annurev-nutr-110420-111710. 34152831 10.1146/annurev-nutr-110420-111710

[3] Hauck C, Cook B, Ellrott TJPotNS. Food addiction, eating addiction and eating disorders. Proc Nutr Soc. 2020;79(1):103–12. 31744566 10.1017/S0029665119001162

[4] Yekaninejad MS, Badrooj N, Vosoughi F, Lin CY, Potenza MN, Pakpour AHJOR. Prevalence of food addiction in children and adolescents: a systematic review and meta‐analysis. Obes Rev. 2021;22(6):e13183. 33403795 10.1111/obr.13183 PMC8244111

[5] de Moraes RCS, Sawaya AL, Vieira ACA, Pereira JKG, de Brito Alves JL, de Luna Freire MO, et al. Food addiction symptoms and metabolic changes in children and adolescents with the double burden of malnutrition. Br J Nutr. 2021;126(12):1911–8. 33494848 10.1017/S0007114521000313

[6] Horsager C, Færk E, Gearhardt AN, Lauritsen MB, Østergaard SD. Food addiction comorbid to mental disorders in adolescents: a nationwide survey and register-based study. Eat Weight Disord. 2022;27(3):945–59. 34089511 10.1007/s40519-021-01212-6

[7] Wang D, Zhou H, Hu Y, Che Y, Ye X, Chen J, et al. Prediction of body fat increase from food addiction scale in school-aged children and adolescents: a longitudinal cross-lagged study. Front Public Health. 2022;10:1056123. 36684883 10.3389/fpubh.2022.1056123 PMC9853519

[8] Stabouli S, Erdine S, Suurorg L, Jankauskienė A, Lurbe E. Obesity and eating disorders in children and adolescents: the bidirectional link. Nutrients. 2021. 10.3390/nu13124321. 34959873 10.3390/nu13124321 PMC8705700

[9] Sehn AP, Silveira JFC, Brand C, Lemes VB, Borfe L, Tornquist L, et al. Screen time, sleep duration, leisure physical activity, obesity, and cardiometabolic risk in children and adolescents: a cross-lagged 2-year study. BMC Cardiovasc Disord. 2024;24(1):525. 39354336 10.1186/s12872-024-04089-2 PMC11443718

[10] Taş Torun Y, İçen S, Gül H, Döğer E. A cross-sectional study on the correlates of food addiction symptoms in adolescents seeking treatment for obesity: eating attitudes and gender differences. J Addict Dis. 2022;40(3):326–35. 34783640 10.1080/10550887.2021.1990638

[11] Borisenkov MF, Tserne TA, Bakutova LA. Food addiction in Russian adolescents: associations with age, sex, weight, and depression. Eur Eat Disord Rev. 2018;26(6):671–6. 30318852 10.1002/erv.2644

[12] Islam MR, Rahman SM, Rahman MM, Pervin J, Rahman A, Ekström EC. Gender and socio-economic stratification of ultra-processed and deep-fried food consumption among rural adolescents: a cross-sectional study from Bangladesh. PLoS ONE. 2022;17(7):e0272275. 35901170 10.1371/journal.pone.0272275 PMC9333446

[13] Oliveira T, Ribeiro I, Jurema-Santos G, Nobre I, Santos R, Rodrigues C, et al. Can the consumption of ultra-processed food be associated with anthropometric indicators of obesity and blood pressure in children 7 to 10 years old? 2020. Foods. 10.3390/foods9111567. 10.3390/foods9111567 PMC7692221 33126771

[14] Popkin BM, Ng SW. The nutrition transition to a stage of high obesity and noncommunicable disease prevalence dominated by ultra-processed foods is not inevitable. Obes Rev. 2022;23(1):e13366. 34632692 10.1111/obr.13366 PMC8639733

[15] Sheen J, Curtin L, Finley S, Konstorum A, McGee R, Craig M. Integrating Diversity, Equity, and Inclusion into preclinical, clinical, and public health mathematical models. Bull Math Biol. 2024;86(5):56. 38625656 10.1007/s11538-024-01282-4 PMC11021228

[16] IBGE. Vitória de Santo Antão 2023. Available from: https://cidades.ibge.gov.br/brasil/pe/vitoria-de-santo-antao/panorama.

[17] Epskamp S, Borsboom D, Fried EI. Estimating psychological networks and their accuracy: a tutorial paper. Behav Res Methods. 2018;50(1):195–212. 28342071 10.3758/s13428-017-0862-1 PMC5809547

[18] Gearhardt AN, Roberto CA, Seamans MJ, Corbin WR, Brownell KD. Preliminary validation of the Yale Food Addiction Scale for children. Eat Behav. 2013;14(4):508–12. 24183146 10.1016/j.eatbeh.2013.07.002 PMC3817415

[19] Association AP. DSM IV: manual diagnóstico y estadístico de los trastornos mentales. DSM IV: manual diagnóstico y estadístico de los trastornos mentales; 1998. p. 907.

[20] Filgueiras AR, Sesso RdCC, Almeida VB, Nogueira PK, Silva CE, Sawaya ALJAeS. Tradução, adaptação e validação preliminar da versão em português do questionário Yale Food Addiction Scale para crianças de baixa renda com excesso de peso. 2019;16(4):46–59.

[21] ABEP. Associação Brasileira de Empresas de Pesquisa. Critério de classificação econômica Brasil. Associação Nacional de Empresas de Pesquisa São Paulo;2022.

[22] Onis Md, Onyango AW, Borghi E, Siyam A, Nishida C, Siekmann J. Development of a WHO growth reference for school-aged children and adolescents. Bull World Health Organ. 2007;85(9):660–7. 18026621 10.2471/BLT.07.043497 PMC2636412

[23] Képes TZ. The critical node detection problem in hypergraphs using weighted node degree centrality. PeerJ Comput Sci. 2023;9:e1351. 37346680 10.7717/peerj-cs.1351 PMC10280579

[24] Xie M, Zhan X-X, Liu C, Zhang Z-K. Influence maximization in hypergraphs. arXiv preprint: arXiv:220601394. 2022.

[25] Leme DEdC, Alves EVdC, Lemos VdCO, Fattoria A. Análise de redes: uma abordagem de estatística multivariada para pesquisas em ciências da saúde. Geriatr, Gerontol Aging (Online). 2020:43–51.

[26] Hu F, Li L, Huang X, Yan X, Huang P. Symptom distribution regularity of insomnia: network and spectral clustering analysis. JMIR Med Inform. 2020;8(4):e16749. 32297869 10.2196/16749 PMC7193440

[27] Ma X, Zhou X, Qiu Y. Bidirectional relationships between cognitive decline and depression: a study of middle-aged and older adults using cross-lagged panel network analysis. J Affect Disord. 2025. 10.1016/j.jad.2025.119741. 40550276 10.1016/j.jad.2025.119741

[28] Rafferty J, Watkins A, Lyons J, Lyons RA, Akbari A, Peek N, et al. Ranking sets of morbidities using hypergraph centrality. J Biomed Inform. 2021;122:103916. 34534697 10.1016/j.jbi.2021.103916 PMC8524321

[29] Kovalenko K, Romance M, Vasilyeva E, Aleja D, Criado R, Musatov D, et al. Vector centrality in hypergraphs. Chaos Solitons Fractals. 2022;162:112397.

[30] Lu H, Uddin S, editors. Disease prediction using graph machine learning based on electronic health data: a review of approaches and trends. Healthcare; 2023: MDPI. 10.3390/healthcare11071031 PMC10094099 37046958

[31] Leary M, Pursey KM, Verdejo-Garcia A, Smout S, McBride N, Osman B, et al. Socio-demographic, self-control, bullying, parenting, and sleep as proximal factors associated with food addiction among adolescents. Behav Sci. 2022. 10.3390/bs12120488. 36546971 10.3390/bs12120488 PMC9774808

[32] Naghashpour M, Rouhandeh R, Karbalaipour M, Miryan M. Prevalence of food addiction among Iranian children and adolescents: associations with sociodemographic and anthropometric indices. Med J Islam Repub Iran. 2018;32:8. 30159259 10.14196/mjiri.32.8 PMC6108267

[33] de Moraes RCS, Viana TAF, Pereira JKG, da Costa PCT, Duarte DB, Toscano LLT, et al. Lower cortisol and dehydroepiandrosterone sulfate and higher food addiction in childhood obesity: associations with stress and dietary parameters. J Endocr Soc. 2025;9(3):bvaf011. 10.1210/jendso/bvaf011 PMC11808062 39931045

[34] Leung CW, Parnarouskis L, Slotnick MJ, Gearhardt AN. Food insecurity and food addiction in a large, national sample of lower-income adults. Curr Dev Nutr. 2023;7(12):102036. 38174213 10.1016/j.cdnut.2023.102036 PMC10761353

[35] Honório OS, Mendes LL, Moreira CC, Araújo ML, Pessoa MC. Evolution of food deserts and food swamps in a Brazilian metropolis between 2008 and 2020. Cien Saude Colet. 2024;29(10):e09582023. 39292043 10.1590/1413-812320242910.09582023

[36] Francis T, Davidson M, Senese L, Jeffs L, Yousefi-Nooraie R, Ouimet M, et al. Exploring the use of social network analysis methods in process improvement within healthcare organizations: a scoping review. BMC Health Serv Res. 2024;24(1):1030. 39237937 10.1186/s12913-024-11475-1 PMC11376022

[37] Filgueiras AR, de Almeida VBP, Nogueira PCK, Domene SMA, da Silva CE, Sesso R, et al. Exploring the consumption of ultra-processed foods and its association with food addiction in overweight children. Appetite. 2019;135:137–45. 30439381 10.1016/j.appet.2018.11.005

[38] Bektas M, Demir Kösem D, Demir Ş, Bektas İ. The effect of food addiction in children on obesity: a systematic review and meta-analysis study. J Pediatr Res. 2021;8(4).

[39] Meseri R, Akanalci C. Food addiction: a key factor contributing to obesity? J Res Med Sci. 2020;25(1):71. 33088308 10.4103/jrms.JRMS_971_19 PMC7554539

[40] Umano GR, Bellone S, Buganza R, Calcaterra V, Corica D, De Sanctis L, et al. Early roots of childhood obesity: risk factors, mechanisms, and prevention strategies. Int J Mol Sci. 2025. 10.3390/ijms26157388. 40806516 10.3390/ijms26157388 PMC12348045

